# Influence of Multi-Frequency Ultrasound-Assisted Freezing on the Freezing Rate, Physicochemical Quality and Microstructure of Cultured Large Yellow Croaker (*Larimichthys crocea*)

**DOI:** 10.3389/fnut.2022.906911

**Published:** 2022-06-16

**Authors:** Xuan Ma, Jun Mei, Weiqiang Qiu, Jing Xie

**Affiliations:** ^1^College of Food Science and Technology, Shanghai Ocean University, Shanghai, China; ^2^National Experimental Teaching Demonstration Center for Food Science and Engineering Shanghai Ocean University, Shanghai, China; ^3^Shanghai Engineering Research Center of Aquatic Product Processing and Preservation, Shanghai, China; ^4^Shanghai Professional Technology Service Platform on Cold Chain Equipment Performance and Energy Saving Evaluation, Shanghai, China

**Keywords:** ultrasound-assisted immersion freezing, large yellow croaker, freezing rate, quality attributes, multi-frequency

## Abstract

The purpose of this work was to investigate the influence of multi-frequency ultrasound-assisted immersion freezing (UIF) on the freezing speed, quality attributes, and microstructure of cultured large yellow croaker (*Larimichthys croce*a) with different ultrasound powers. The findings revealed that UIF under multi-frequency conditions greatly enhanced the speed of food freezing. The multi-frequency UIF reduced the thawing and cooking losses, total volatile base nitrogen, *K*-values, and thiobarbituric acid reactive substances values, and increased the water holding capacity. The microstructure observation showed that multi-frequency UIF at 175 W reduced pore diameter and ice crystal size. Free amino acids analysis revealed that the application of multi-frequency UIF reduced the accumulation of bitter amino acids, and UIF-175 treatment increased the accumulation of umami amino acids. Therefore, multi-frequency UIF at a suitable ultrasonic power can remarkably improve the quality of large yellow croaker.

## Introduction

Fresh fish are prone to spoilage because of their high moisture activity, enzyme activity, and abundant nutrients (1). Freezing is one of the most commonly used methods to reduce food spoilage and extend their shelflife by limiting microbial activity and lowering the biochemical processes (2). The freezing rate could affect the quality of frozen products. Fast freezing helps to form a large quantity of fine, evenly dispersed ice crystals inside and outside the cell, which lead to less injury to the muscle structure and retard the reduction of fish juices after thawing (3). Accordingly, fast freezing is a good way to keep the quality of frozen fish and fish products (4).

To improve the freezing process, several freezing techniques have been implemented, among them UIF has been demonstrated to be an efficient way to shorten the freezing time (5). UIF can produce abundant cavitation bubbles, which function as crystal nuclei and facilitate the process of ice crystal formation (6). The collapse and propagation of the cavitation bubbles produce intense forces, namely, microstreaming, to disintegrate the large ice crystals that already existed into much smaller fractions that can also be used as new crystal nuclei to facilitate secondary ice nucleation (7, 8). Furthermore, the convective heat transfer rate is effectively increased by macroscopic turbulence induced by ultrasound (9). The heat and mass transfer are also improved by the fast movement of the cavitation bubbles (6). Tu et al. (10) suggested that UIF sped up the freezing process of lotus roots and its samples exhibited an enhanced firmness, decreased drip loss, and caused less microstructural disruption. Cheng et al. reported that ultrasound intensity when applied at a higher magnitude was highly effective in shortening the characteristic freezing time of strawberries (11). Islam et al. found that ultrasound-assisted immersion freezing significantly reduced the nucleation time and total freezing time during immersion freezing of mushrooms (12). Xu et al. (5) reported that ultrasound application can shorten freezing time and drip loss of red radish and show better retention of firmness and color. Xin et al. (13) reported that the freezing time was reduced and the microstructure and physical and chemical qualities of broccoli were better preserved by UIF. Thus, UIF aids in the preservation of frozen food quality by speeding up the freezing process and facilitating the creation of fine and regular-shaped ice crystals (14). Besides, Islam et al. pointed out that the ultrasound shock waves produce cavitation and acoustic streaming in the liquid medium, which generates adequate mechanical, thermal, and chemical effects to inactivate enzymes and microbes and enhances the ice crystal nucleation process (15, 16). The rise in mechanical interference and cavitation nuclei of multi-frequency ultrasonic equipment can boost cavitation yields more than traditional ultrasonic cleaners or single-frequency ultrasonic equipment (17). According to the above findings, we supposed that multi-frequency ultrasound could decrease the impairment of fish throughout the freezing process. Nevertheless, there is virtually little information on the influence of multi-frequency UIF with different powers on the quality characteristics of large yellow croaker. Consequently, the purpose of the present work was to investigate the impact of multi-frequency UIF with various power on the freezing speed, physicochemical attributes, and microstructure in large yellow croaker (*Larimichthys crocea*) to improve the quality of frozen fish and reduce the freezing injury.

## Materials and Methods

### Freezing Treatments

**Figure 1:**
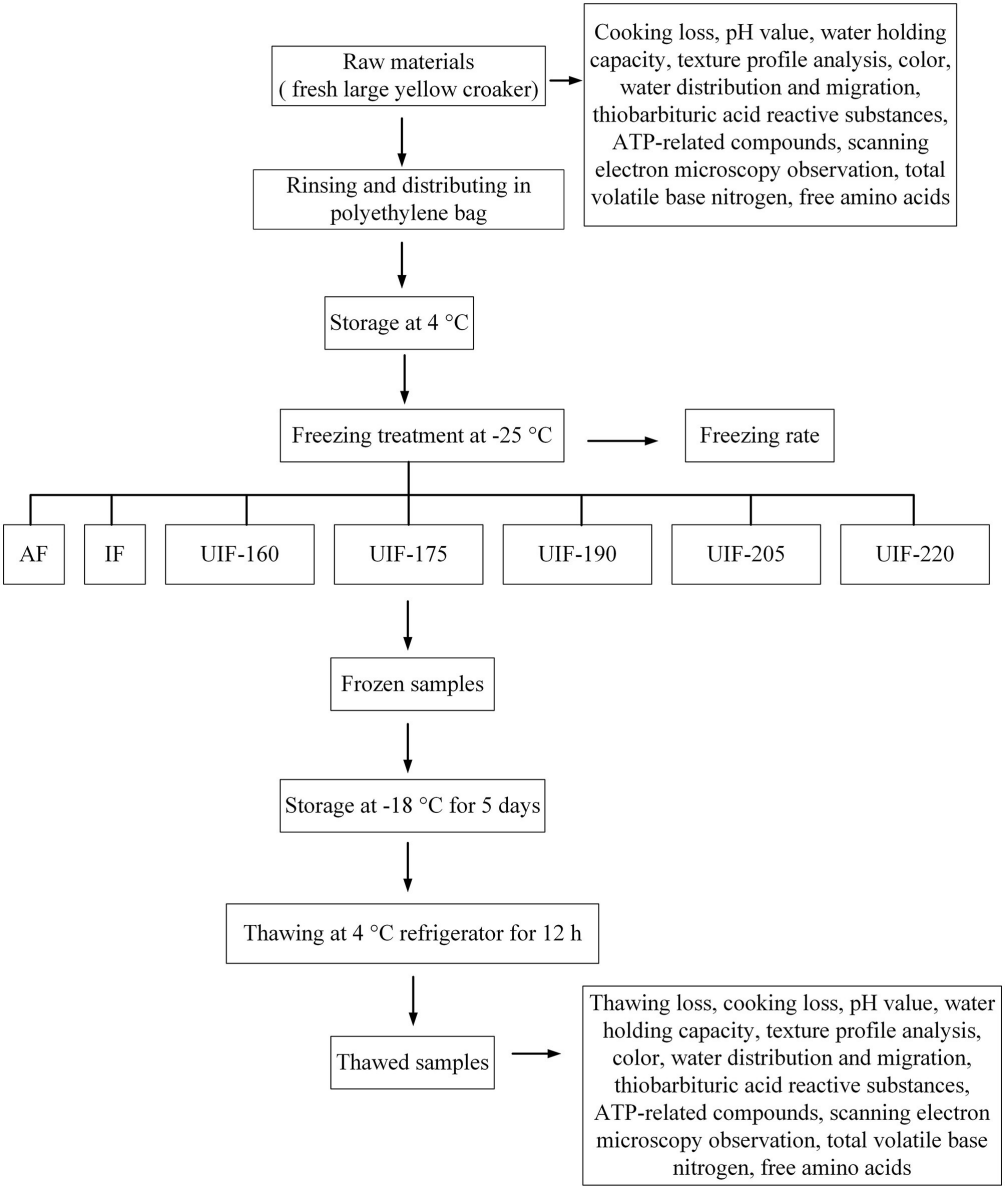
Schematic diagram showing the experimental procedure.

Fresh cultured large yellow croaker (~500 g) were acquired from the local market in Luchao Port town (Shanghai, China) and kept at 4°C to ensure the same initial sample temperature before the freezing process. The fresh fish were frozen with air freezing (AF), immersion freezing (IF), and UIF. The UIF was performed in an ultrasonic bath instrument with triple-ultrasound frequencies at 20, 28, and 40 kHz. The ultrasonic bath instrument was the same as that described by Ma et al. (18) with 29.3% calcium chloride brine solution (w/v) as the freezing medium. The AF-treated samples were frozen at −25.0°C in a freezer. For IF and UIF treatments, the large yellow croaker samples were fully immersed in the freezing medium at −25°C. The treatments (IF, UIF-160, UIF-175, UIF-190, UIF-205, and UIF-220 referring to samples applied with 0, 160, 175, 190, 205, and 220 W, respectively), were set to run for 9 min with an interval of 30 s, until the center temperature reached −18°C. A T-type thermocouple connected to a temperature collection device (Fluke 2640A, Fluke Electronic Instrument Co., Ltd, United States) was placed into the samples to test and collect the central temperature. The frozen fish samples were maintained at −18°C for 5 days until subsequent determinations. Figure 1 reveals the schematic diagram of the experimental procedure.

### Thawing Loss

The thawing loss was tested by the methods of Li et al. (19). The samples were thawed with cooled running water until the central temperature reached 4°C. The water on the surface was absorbed by a filter paper. The thawing loss was acquired through the following equation:


(1) $$\begin{array}{r} \text{Thawing loss }\left(\text{\%}\right)=\frac{{\text{W}}_{0}-{\text{W}}_{1}}{{\text{W}}_{0}}\times 100\text{ \%} \end{array}$$

W_0_: the weight before thawing (W_0_);

W_1_: the weight following thawing (W_1_).

### Cooking Loss

The cooking loss was measured by the methods of Hong et al. (20). The thawed fish samples were cut into 3 × 3 × 1 cm pieces and packaged separately in polyethylene bags. Then they were cooked at 75°C in a water bath for 15 min and the water on the surface was absorbed by a filter paper. The cooking loss was acquired through the following equation:


(2) $$\begin{array}{r} \text{Cooking loss }\left(\text{\%}\right)=\frac{{\text{W}}_{2}-{\text{W}}_{3}}{{\text{W}}_{2}}\times 100\text{ \%} \end{array}$$

W_2_: the weight prior to cooking (W_2_);

W_3_: the weight following cooking (W_3_).

### pH Value

Fish samples (5 g) were mixed and then added to 45 ml of deionized water and centrifuged at 3,040 × g for 5 min by centrifuge (H-2050R, Xiangyi Centrifuge Co., Ltd, Hunan, China), according to the methods of Tan et al. (21). The pH value of the supernatant was determined using a pH meter (PB-10, Sartorius, Germany).

### Water Holding Capacity

The WHC was estimated according to Luan et al. (22). Briefly, 2 g of fish muscle were placed in the centrifuge tube and spun at 3,040 × g for 10 min at 4°C. After centrifugation, the samples were taken out of the tubes and the weight differences were calculated. The weight difference was shown as a percentage of the initial weight of the sample.

### Texture Profile Analysis

Texture profile analysis (TPA) of thawed dorsal muscle for each treatment was performed using the TA. XT Plus texture analyzer (Godalming, Surrey, UK) with a P/5 probe (23). The hardness, chewiness, springiness, and cohesiveness were determined. The test speed was 1 m/s, and the sample deformation was 50%. A minimum of 6 points were tested for each sample.

### Color

The color of fish samples after thawing and fresh samples was determined by a colorimeter (CR-400, Konica Minolta, Tokyo, Japan) following the methods of Suemitsu and Cristianini (24). The colorimeter was checked with a white reference tile for calibration before each measurement. The determination was carried out at different points to analyze the LAB values for each sample.

### Water Distribution and Migration

The sample dorsal muscle was chopped into 3 × 2 × 2 cm sections covered with polyethylene film and determined by a low-field nuclear magnetic resonance (LF-NMR) analyzer (Niumag MesoMR23-60H.I, Suzhou, China) following the methods introduced by Liu et al. (25). The critical parameters are as shown below: the receiver bandwidth frequency (SW) = 100 kHz, the parameter to control the first data point that acquired (RFD) = 0.08, the number of the scans (NS) = 4, P1 = 19 μs, P2 = 37 μs, analog gain (RG1) = 20 dB, digital gain (DRG1) = 6 dB, preamplifier gain (PRG) = 1, and the duration between successive scans (TW) = 2,000 ms. Magnetic resonance imaging experiments of large yellow croaker samples were also performed to get proton density-weighted images. The parameters of the collection are as follows: slice width = 1.5 mm, time repetition = 500, and time echo = 20 ms. Three photographs were taken for each group of samples.

### Thiobarbituric Acid Reactive Substances

Thiobarbituric acid reactive substances (TBARS) determination was performed in accordance with the method proposed by Li et al. (26). Five grams of flesh and 20 ml of 20% TBA solution were homogeneously mixed and allowed to stand for 1 h. Then, the mixture was centrifuged at 11,960 × g at 4°C for 10 min, and the supernatants were collected. Five milliliters of collected supernatant was mixed with 5 ml of thiobarbituric acid (TBA, 0.02 M) and boiled for 40 min. Then the mixture was immediately transferred to an ice bath, and the absorbance was measured at 532 nm. The value of TBARS was represented by the MDA content. It was calculated by the following formula and expressed as mg MDA/kg. The absorbance of the sample and the standard was denoted by A_1_ and A_0_, respectively, and the mass fraction was ω_0_.


(3) $$\begin{array}{r} \text{TBARS}\left(\text{mg MDA}/\text{kg}\right)=\frac{{\text{A}}_{1}}{{\text{A}}_{0}}\times {\text{ω}}_{0} \end{array}$$

### ATP-Related Compounds

ATP-related compounds containing IMP, HxR, and Hx were extracted with 0.6M perchloric solution. The extraction mixture was centrifuged at 5,980 × g for 10 min, and then the pH was adjusted to 6.5–6.8. After filtration through a 0.22-μm filter, the ATP-related compounds were determined by HPLC (Waters 2695, Milford, United States) following the methods introduced by Karim et al. (27). HPLC was conducted under the following conditions: Waters 2695 liquid chromatograph, Shim-pack VP-ODS C18 column (46 × 150 mm), the mobile phase was a mixture of phosphate buffer solution (20 mmol/L potassium dihydrogen phosphate/dipotassium hydrogen phosphate, pH 6.7), methanol solution (95:5, v/v), at a rate of 1.0 ml/min. The loading amount was 10 μl. The K value was calculated by the equation:


(4) $$\begin{array}{l} \text{ K value }\left(\%\right)\text{ = }\\ \frac{\text{HxR + Hx}}{\text{ATP + ADP + AMP + IMP + HxR + Hx }}\times \text{100} \end{array}$$

ATP, ADP, AMP, IMP, HxR, and Hx represent adenosine triphosphate, adenosine diphosphate, adenosine monophosphate, inosine monophosphate hypoxanthine, riboside, and hypoxanthine, respectively.

### Scanning Electron Microscopy Observation

The frozen dorsal muscle samples (~3 × 3 × 2 mm) were freeze-dried and electroplated with a gold conductive coating. The microstructure was determined by Extreme-resolution Analytical Field Emission SEM (Mira 3 XH, Tescan, Brno, Czech Republic) with an accelerating voltage of 5 kV, and the microstructure was examined at 250 × magnification (28).

### Total Volatile Base Nitrogen

Total volatile base nitrogen (TVB-N) was measured using the microtitration method following the method proposed by Cao et al. (29) with appropriate modifications. A 5 g of fish sample was weighed accurately and ~2 g of MgO powder was added. TVB-N was examined using Kjeldahl apparatus (Kjeltec8400, Foss, Hilleroed, Denmark). The amount of TVB-N was calculated using the following equation:


(5) $$\text{TVB-N}(\frac{\text{mg N}}{\text{100 g}})\text{ = }\frac{({\text{V}}_{1}\text{ - }{\text{V}}_{2})\times \text{C}\times \text{14}}{\text{m}}\times \text{100}$$

Where V_1_ is the volume of the HCl used for the titration of sample (ml), V_2_ is volume of the HCl for the titration of blank (ml), C is the concentration of HCl (mol/L), and m is the weight of fish samples (g). The results of TVB-N values were presented as mg N/100 g large yellow croaker samples. Three parallel experiments were performed and the average value was obtained.

### Free Amino Acids

Free amino acids (FAAs) were determined using the method proposed by Li et al. (30). A 2 g of chopped large yellow croaker sample and 10 ml of 5% cold trichloroacetic acid were mixed and homogenized at 10,000 × g for 10 min. The extraction and centrifugation were repeated and the combined supernatants were diluted to 25 ml. The supernatant was filtered through a 0.22-μm filter. An automatic amino acid analyzer (Hitachi L-8800, Tokyo, Japan) was used for evaluation. The quantification of FAAs was accomplished automatically by the installed program according to the retention times and peak areas of FAAs standards (Sigma Chemical Co. St Louis, MO, United States). Three replicate measurements were carried out.

### Statistical Analysis

The results of the experiment were statistically evaluated using SPSS 22.0 software and reported as mean ± standard deviation. Significant differences (*p* < 0.05) in treatment means were determined using one-way ANOVA and Duncan's multiple range test.

## Results and Discussions

### Freezing Rate

**Figure 2:**
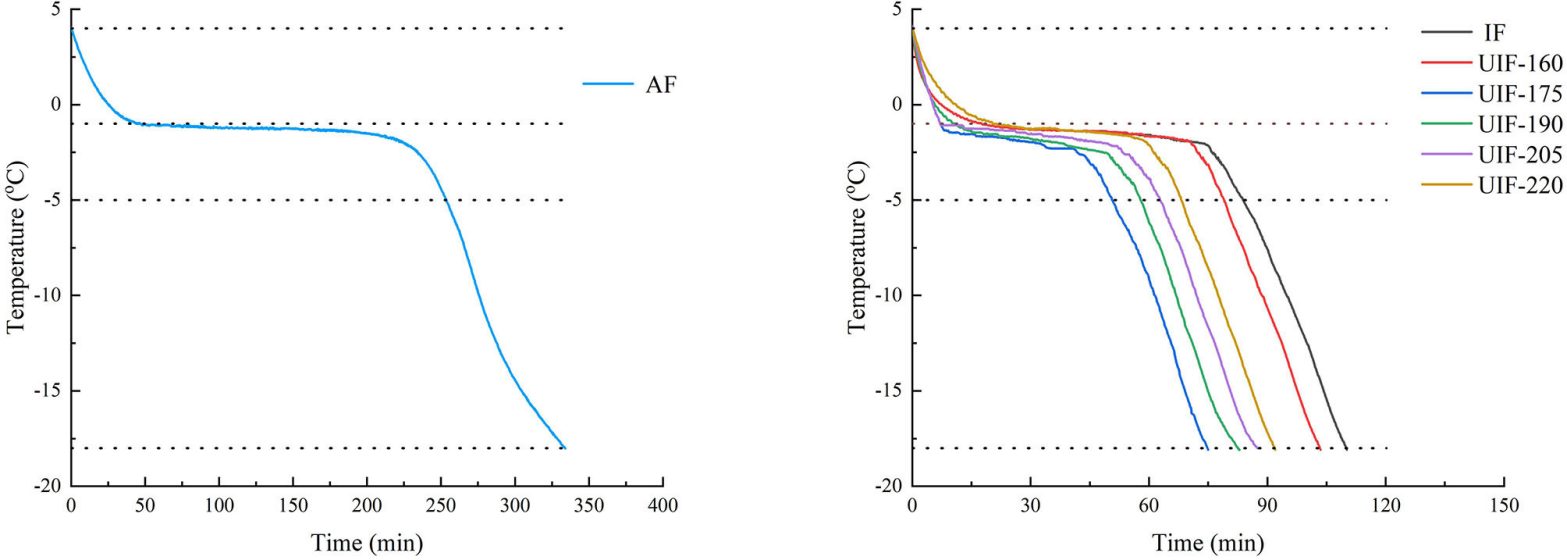
Freezing curves of large yellow croaker with different freezing treatments.

Temperature change curves throughout the freezing process were used to visually depict the effect of various freezing treatments on large yellow croaker freezing time (Figure 2). The total freezing time of large yellow croaker samples in this study referred to the time required for the temperature to drop from 4 to −18°C. The total freezing time for the AF-treated samples was 333.83 ± 2.38 min, more than 3 times the time required for the IF-treated samples (*p* < 0.05). Since the heat transfer coefficient in liquid media is much higher than that in air, IF treatment can substantially enhance the freezing rate and shorten the total freezing time (31). When ultrasound was supplied, the freezing time was first reduced and then increased along with the increasing ultrasonic power. The UIF-175-treated samples had the shortest total freezing time, which was 29.71% less than the IF-treated samples (*p* < 0.05). This occurrence could be attributed to numerous cavitation bubbles generated by UIF-175 treatment functioning as nuclei, facilitating the production of ice crystals and therefore reducing the freezing period. The results demonstrated that UIF at suitable powers remarkably enhanced the freezing speed of large yellow croaker as compared to IF.

The freezing process was classified into three steps, namely, pre-cooling stage, phase transition stage, and sub-cooling stage (18). There was no significant difference in the pre-cooling time found between samples with IF and UIF treatments (*p* > 0.05) during the pre-cooling phase, without the application of ultrasound (Table 1). In the phase change stage, most of the water would crystallize, so this stage can directly determine the freezing quality of the product (32). As the ultrasonic power became higher, the phase transition time increased and then diminished, which was consistent with the results of the total freezing time. The UIF-175-treated samples displayed the minimum phase transition time, which is 34.26% shorter than that of IF (*p* < 0.05). This trend may be related to the increase in convective heat transfer rate caused by the microstreaming and cavitation effect (33). Zhang et al. (34) reported that UIF increased the freezing speed of chicken breast, and 165 W of ultrasonic power (UIF-165) diminished the freezing time. Sun et al. (35) found that the freezing period of common carp was dramatically reduced when UIF was used at the suitable ultrasonic power. Sun and Li (36) also indicated that the use of ultrasound increased the freezing rate of potato samples when compared to no ultrasound. These investigations demonstrated that UIF could increase the freezing rates of food materials. The fastest heat transfer rate was observed at an ultrasonic power of 175 W in this work. The phase transition time was increased (*p* < 0.05) at a comparatively high level of ultrasonic power (UIF-205 and UIF-220), and the corresponding freezing rate exhibited a decrease compared to UIF-175. The high-intensity ultrasonic power generates excessive heat on the surface of the sample as a barrier to heat transfer between the muscle and the coolant (6). The higher ultrasonic power can negatively affect the freezing efficiency at this time. The thermal conductivity increased during the subcooling stage since a large quantity of water inside the muscle froze, and the temperature dropped quickly in comparison with the phase transition stage. The AF-treated samples showed the highest subcooling time, but there was no significant difference between IF- and UIF-treated samples (*p* > 0.05).

**Table 1:** Influence of different freezing methods on the freezing time of the large yellow croaker.

**Treatment**	**Pre-Cooling stage (min)**	**Phase transition stage (min)**	**Sub-cooling stage (min)**	**Total freezing time (min)**
AF	46.33 ± 1.27^a^	207.24 ± 2.02^a^	80.50 ± 1.67^a^	333.83 ± 2.38^a^
IF	17.83 ± 1.75^b^	66.17 ± 1.35^b^	26.17 ± 1.02^b^	110.72 ± 0.95^b^
UIF-160	17.67 ± 0.62^b^	61.50 ± 1.66^c^	24.16 ± 0.94^b^	104.73 ± 1.17^c^
UIF-175	10.33 ± 0.16^b^	43.50 ± 1.32^d^	24.17 ± 1.26^b^	77.83 ± 0.97^e^
UIF-190	10.67 ± 0.23^b^	47.33 ± 1.44^d^	24.50 ± 1.05^b^	82.67 ± 1.42^d^
UIF-205	12.67 ± 0.48^b^	50.33 ± 1.27^e^	24.50 ± 0.79^b^	87.78 ± 2.03^d^
UIF-220	18.26 ± 1.41^b^	52.83 ± 2.15^e^	24.74 ± 2.13^b^	95.92 ± 1.37^f^

*The means in the same column with different superscript letters differ significantly (p < 0.05). The results are mean ± standard deviation (n = 3)*.

### Thawing Loss, Cooking Loss, and WHC Analysis

**Figure 3:**
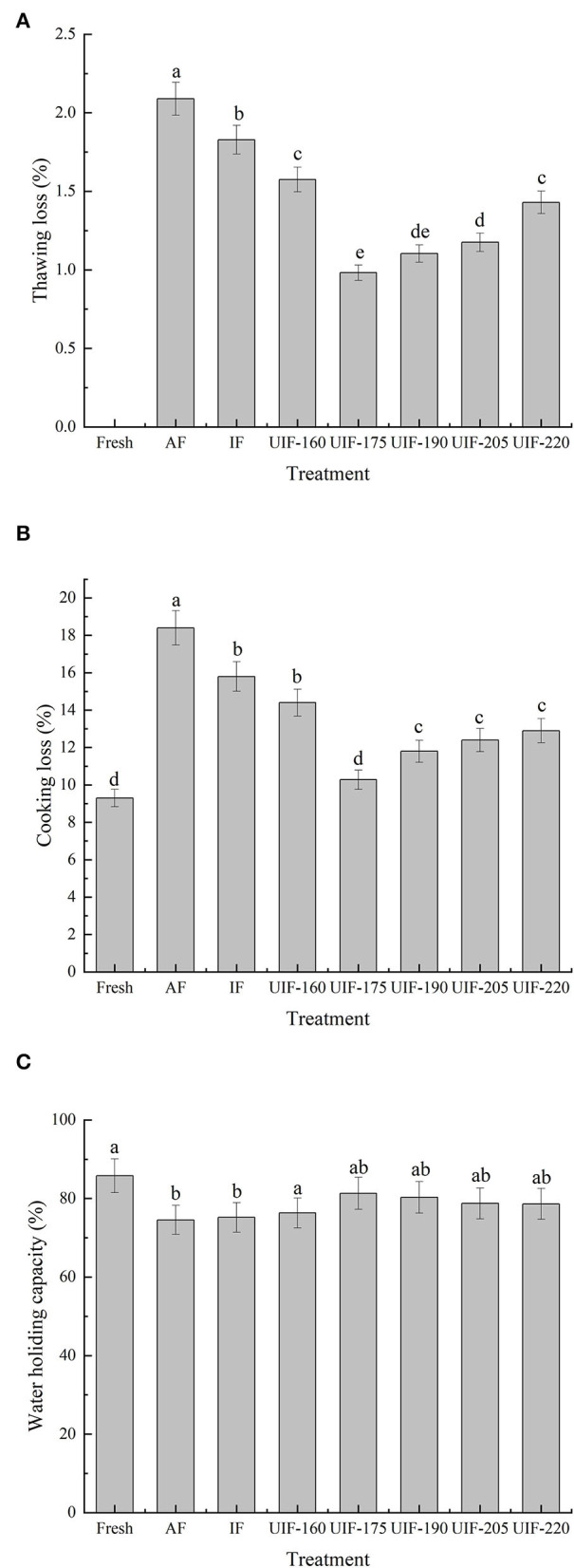
Influence of different freezing treatments on the thawing loss **(A)**, cooking loss **(B)**, and WHC **(C)** of the large yellow croaker. The results are average ± standard deviation (*n* = 3).

The effects of several freezing processes on the thawing loss, cooking loss, and WHC of large yellow croaker are displayed in Figure 3. The appearance, weight, and organoleptic characteristics of fish and fish products can be affected by thawing loss. Thawing loss in AF-treated samples was 2.09 ± 0.10%, while it was 1.83 ± 0.09% in those treated with IF (Figure 3A). As the ultrasonic power gradually increases, the thawing loss revealed a trend of decreasing and then rising. Samples treated with ultrasonic power at 175 W exhibited the minimum thawing loss, which was 53.11 % lower than the AF-treated samples and 46.45 % lower than the IF-treated samples (*p* < 0.05). Sun et al. (37) compared the influence of UIF, AF, and IF on the muscle quality of common carp during freezing storage and found that lower thawing loss was achieved after UIF (175 W, 30 kHz) compared to air and immersion freezing materials. Xin et al. (38) and Islam et al. (12) reported alike findings. Quick freezing speed provided by suitable ultrasonic power contributed to more tiny ice crystals equally distributed throughout the muscle, resulting in decreased thawing loss (5). For AF- and IF-treated samples, endocellular water slowly flowed to the exterior cellular region, forming larger exterior cellular ice crystals, which can result in significant tissue distortion and cell shrinkage (18).

**Figure 4:**
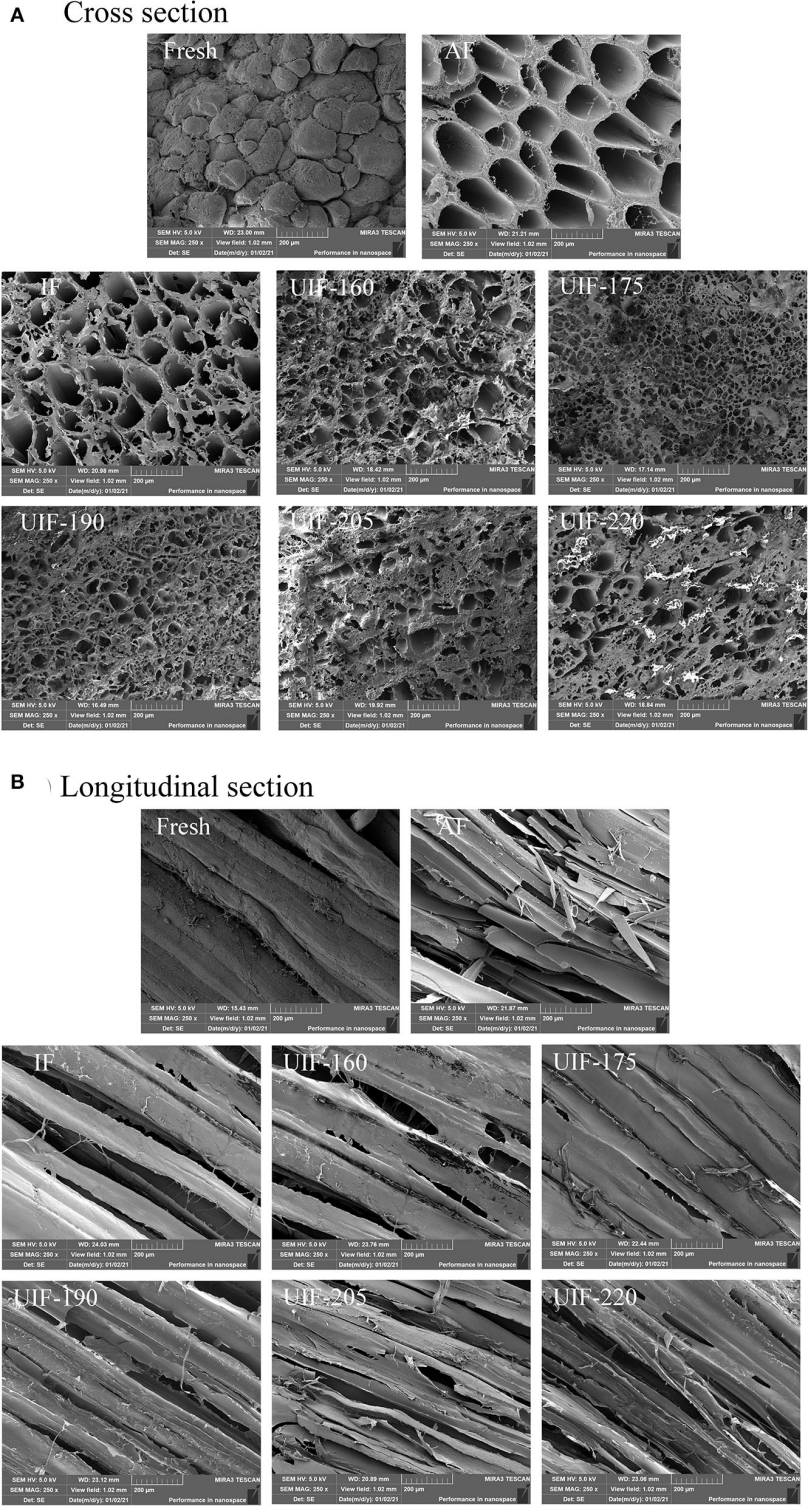
Influence of different freezing treatments on the microstructure of the large yellow croaker. **(A)** Cross section and **(B)** Longitudinal section.

During the cooking process, a mixture of liquid and soluble components from the muscle is normally lost (20). The cooking losses of the samples followed the same tendency as the thawing losses. The data presented in Figure 3B show that AF-treated samples had the highest cooking loss (18.40 ± 0.93 %), followed by IF-, and UIF-treated samples, whereas no significant differences were observed among the fresh sample and the samples treated with UIF-175 (*p* > 0.05). The UIF-175-treated samples had the lowest cooking loss (10.28 ± 0.51 %) among the UIF-treated samples and was nearest to the fresh sample with a cooking loss of 9.30%. This tendency can be explained by the large number of ice crystals created through slow freezing (AF) that disrupts the muscle structure, which caused a reduction in WHC of muscle tissue and an increase in cooking loss (Figure 4). The UIF-175-treated samples contained homogenous and small ice crystals, as revealed in muscle microstructures (Figure 4), which successfully protect the integrity of muscle tissue throughout the freezing process and lead to lower cooking loss. Islam et al. reported that ultrasound irradiation initiated the nucleation of ice and reduced the mean size of ice crystals during freezing and frozen storage, and therefore improved the frozen product quality compared to the control samples (39).

The sensory qualities of fish muscles are highly associated with WHC. The AF samples had the lowest WHC (74.56 ± 3.73%), as followed by the IF- and UIF-treated samples (Figure 3C). Especially, the UIF-175-treated samples had the highest WHC (81.33 ± 4.07%) and were comparable to the fresh samples with a WHC of 85.78 ± 4.29% (*p* > 0.05). In the current study, UIF's ability to increase WHC is due to its capacity to change or mechanically disrupt the native protein structure, exposing more active groups such as reactive sulfhydryl and hydrophobic groups, and the formation of small spaces with uniform structure between myofibrillar proteins, which can hold the water molecules firmly and reduce water loss when centrifuged (40).

### Scanning Electron Microscope

The microstructure of muscle has a great impact on fish quality. To determine how different freezing treatments altered the size and distribution of ice crystals, cross-sections (Figure 4A) and longitudinal sections (Figure 4B) of frozen muscle tissue were studied. The fresh samples showed finer and serried pores than other samples. Because the AF samples had a slower freezing speed than the other samples, they had the largest pore size. This resulted in the production of numerous large crystals inside and outside the cells. The size of the pores is also associated with the size and distribution of the ice crystals, which are both influenced by the freezing rate. The IF samples contained considerably smaller holes than the AF samples, suggesting that smaller ice crystals were formed. The UIF-175 samples revealed the minimum and most homogeneous ice crystals, which can be demonstrated by having a high density of small pores. This result suggested the less structural damage of UIF-175 samples, which explains the lower thawing and cooking losses mentioned earlier. As a consequence, appropriate ultrasonic power can diminish the size of the ice crystal. Islam et al. applied solid–solid contact ultrasound to the freezing and frozen storage of mushrooms and the results showed that ultrasound accelerated the nucleation of ice crystals and reduced the size distribution of ice crystals (39). Saclier et al. (41) suggested that cavitation bubbles and microstreaming produced by ultrasound-induced nucleation split pre-existing ice crystals into smaller nuclei and facilitated secondary nucleation, leading to more ice nuclei and a faster freezing rate. Islam et al. (42) found that with the application of power ultrasound, dried samples showed more porous structures and the free volume between the cells increased. This may be because acoustic cavitation caused by the ultrasonic power produces small vapor-filled bubbles that collapse rapidly. Such collapse of bubbles led to the completed and accelerated degassing of immersed solid. However, insufficient (UIF-160) or excess (UIF-205 and UIF-220) ultrasonic power resulted in larger ice crystals, which was relevant to the freezing time (43). The microstructure of large yellow croaker frozen with ultrasound was better than samples frozen without ultrasonic treatment. Under the condition of high ultrasonic power, the destructive role of ultrasonic cavitation, as well as the vibration of the ultrasound can result in muscle physical structure to degrade. Zhang et al. (44) found a similar phenomenon that further increases in ultrasonic power from 180 to 240 and 300 W resulted in an increase in ice crystal size and wider gaps between muscle fibers, resulting in bigger structural damage to the samples.

### Texture Profile Analysis

**Figure 5:**
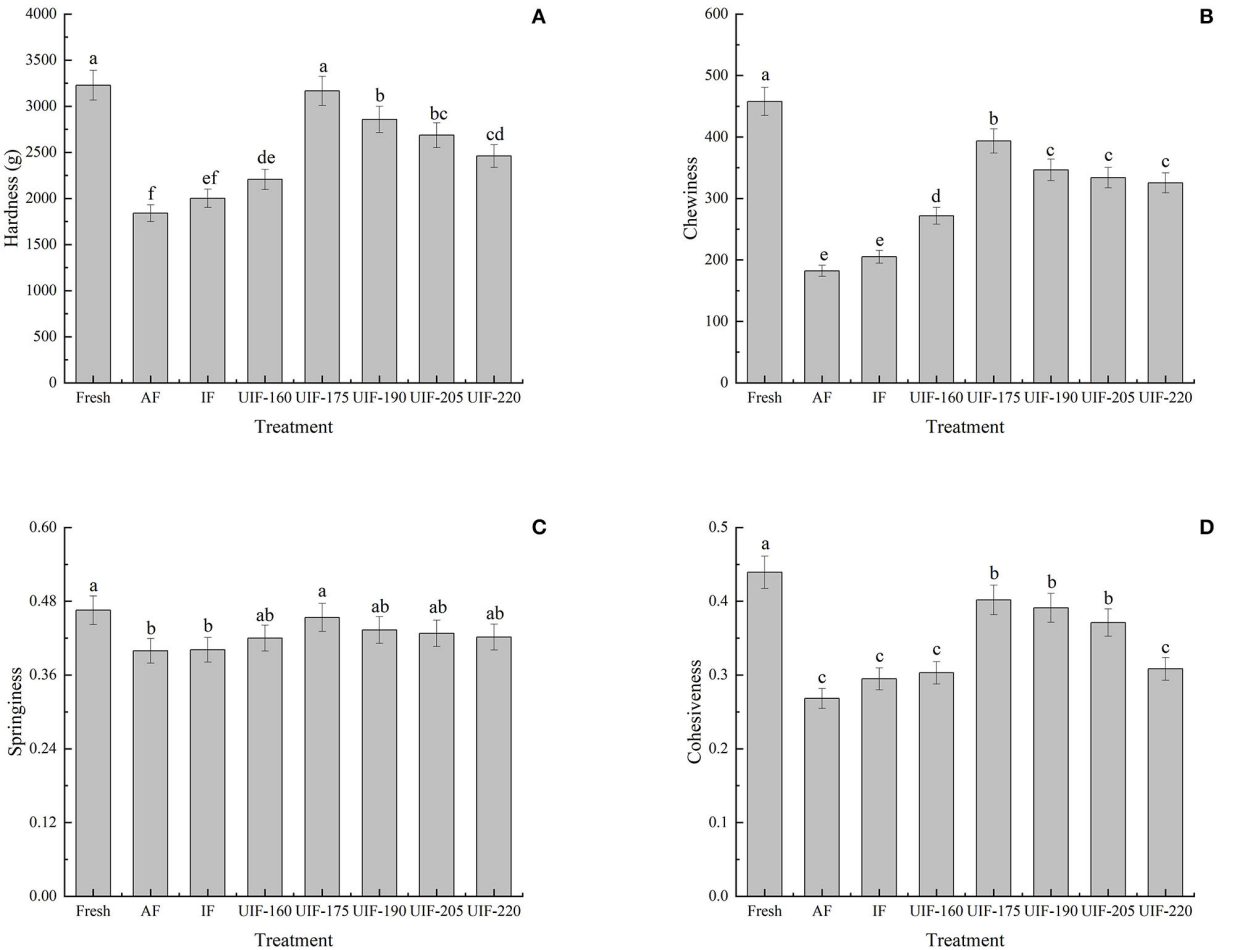
Influence of different freezing treatments on the hardness **(A)**, chewiness **(B)**, springiness **(C)**, and cohesiveness **(D)** of the large yellow croaker. The results are average ± standard deviation (*n* = 3).

The textural qualities of large yellow croaker samples were analyzed by hardness, chewiness, springiness, and cohesiveness. Different ultrasonic power displayed an important influence on the muscle hardness variation of samples, as shown in Figure 5A. The AF-treated sample showed the minimum hardness (1,840.03 ± 92.17 g). The UIF-175-treated samples exhibited the maximum hardness (3,167.56 ± 158.78 g) and were nearest to that of the fresh sample (3227.88 ± 161.39 g). No significant differences were observed between the fresh sample and the samples treated with UIF-175 (*p* > 0.05). Ultrasonic treatment revealed higher chewiness, springiness, and cohesiveness of the fish dorsal muscle than both AF and IF treatments (Figures 5B–D), where the samples of UIF-175 showed greater chewiness (393.54 ± 19.68), springiness (0.45 ± 0.02), and cohesiveness (0.40 ± 0.03) than other ultrasonic-treated samples. A significant difference was observed in hardness and chewiness among the samples with UIF-175 treatments and other ultrasonic-treated samples (*p* <0.05). The well-textural properties in the samples treated with UIF-175 can be connected with the development of fine and uniform ice crystals, thereby reducing structural impairment to the muscle throughout the freezing process. In addition, Wang et al. (45) proposed that better textural qualities could also be attributed to the increase in WHC, which is in agreement with the findings in Figure 3C.

### Color and pH Value

Color is a property directly utilized for evaluating visual quality and plays a vital part in the appearance and acceptability of frozen meat (46). All of the frozen samples had greater L^*^ values than the fresh sample (Table 2). The L^*^ values in the AF-treated samples were apparently higher (*p* < 0.05) than that in the fresh sample. Since AF-treated samples exhibit a higher thawing loss, there was more free water on the surface of fish, contributing to a larger L^*^ value. According to the findings of Farouk et al. (47), the color of slowly frozen and thawed beef is lighter than that of rapidly frozen beef. A larger quantity of thawing loss, according to Muela et al. (48), may produce more light reflection and lighter color in slowly frozen samples, which was consistent with the thawing loss discussed above. In comparison with AF- and IF-treated samples, L^*^ values were significantly reduced in UIF-treated samples (*p* < 0.05). Among the ultrasonic treatment samples, as the ultrasonic power became higher, the L^*^ value first declined and then increased. The UIF-175-treated samples, in particular, exhibited the lowest L^*^ value and were nearest to that of the fresh sample. L^*^ values are subject to structural changes in the muscle caused by water outflow from the cells throughout the freezing process. Meat structure changes such as disruption of protein conformations can increase light dispersion, thus increasing the L^*^ value of meat (49). Furthermore, as a lot of water went outside the cells, a higher-intensity ultrasound could raise the L^*^ values of the muscles. Pohlman et al. (50) stated that a high-intensity ultrasound treatment at 22 W/cm^2^, 20 kHz increased the L^*^ value of beef pectoralis muscle. However, a^*^ or b^*^ values of large yellow croaker were not significantly affected by various freezing processes (*p* > 0.05). Lind et al. (51) found a similar phenomenon that the freezing speed showed no significant impact on the a^*^ values in lamb, probably because myoglobin had recovered its native conformation and regained its color after thawing of the samples.

**Table 2:** Influence of different freezing treatments on the color and pH of large yellow croaker.

**Treatment**	**Color**			**pH**
	L*	a*	b*	
Fresh	46.45 ± 1.51^c^	3.05 ± 0.48^a^	2.61 ± 0.66^a^	7.22 ± 0.01^c^
AF	58.46 ± 1.22^a^	3.57 ± 0.42^b^	2.74 ± 0.55^a^	7.02 ± 0.07^a^
IF	56.24 ± 1.68^a^	3.50 ± 0.26^b^	2.34 ± 0.64^a^	7.10 ± 0.01^ab^
UIF-160	54.34 ± 1.16^b^	3.77 ± 0.99^b^	2.47 ± 0.24^a^	7.11 ± 0.04^b^
UIF-175	47.19 ± 0.95^bc^	3.55 ± 0.88^b^	2.59 ± 0.80^a^	7.17 ± 0.03^b^
UIF-190	50.96 ± 1.07^b^	3.43 ± 0.17^b^	2.64 ± 0.92^a^	7.13 ± 0.02^b^
UIF-205	52.20 ± 0.63^bc^	3.63 ± 1.12^b^	2.74 ± 0.43^a^	7.09 ± 0.01^b^
UIF-220	52.38 ± 1.31^bc^	3.18 ± 0.10^b^	2.68 ± 0.64^a^	7.13 ± 0.03^b^

*The means in the same column with different superscript letters differ significantly (p < 0.05). The results are average ± standard deviation (n = 3)*.

The pH value can have an impact on the quality of fish, which indicates the degree of deterioration in fish. AF-, IF-, and UIF-treated samples revealed a comparatively lower pH value than fresh samples (Table 2), which can be attributed to the accumulation of inorganic phosphoric acid and lactate as well as deterioration of adenosine triphosphate throughout the freezing and thawing processes (52). But no significant differences were found among samples with IF and UIF treatments (*p* > 0.05). Generally, pH changes were primarily dependent on the levels of ammonia, organic sulfides, and amines resulting from the degradation of proteins by microorganisms and endogenous enzymes (48). The short freezing time did not lead to remarkable variations in pH for this study. In addition, Zhang et al. (44) suggested that it is not the pH that induced the changes in thawing and cooking losses. Similar results were reported by Sun et al. (35), who assessed the influence of UIF on the common carp quality and found no significant differences (*p* > 0.05) in the pH of the fish samples at various UIF conditions.

### Low-Field Nuclear Magnetic Resonance

**Figure 6:**
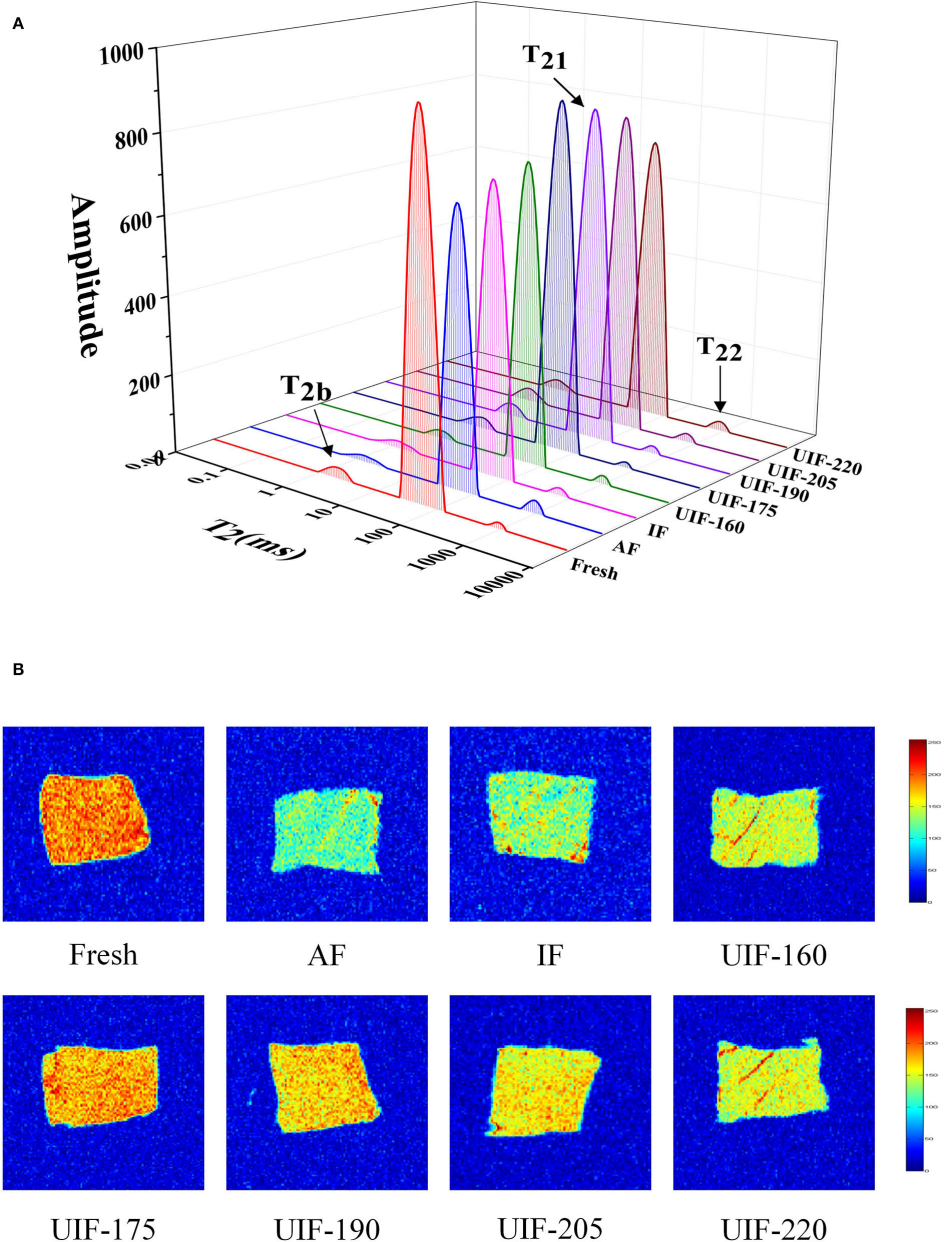
Influence of different freezing treatments on the water distribution **(A)** and magnetic resonance imaging **(B)** of the large yellow croaker.

LF-NMR can be applied to evaluate the distribution and fluidity of moisture inside the frozen muscle under various freezing conditions. Three different water populations were identified by three peaks (Figure 6A). T_2b_ (0–10 ms) denotes bound water, T_21_ (10–100 ms) represents immobilized water, and T_22_ (100–1,000 ms) represents free water (37).

The pT_2b_, pT_21_, and pT_22_ corresponded to the areas of T_2b_, T_21_, and T_22_, respectively. There were no obvious variations among the fresh, AF, IF, and UIF samples in pT_2b_ relaxation (Figure 6A), indicating that UIF had no apparent negative influence on the damaged capillaries of large yellow croaker. Zhang et al. (53) reported no significant change (*p* > 0.05) in the T2_b_ relaxation time of the longest porcine muscle after one freeze–thaw cycle. This is owing to the existence of bound water in the muscle tissue, which is unaffected by mechanical stress or microstructural changes. The amount of bound water does not fluctuate much, and it is extremely resistant to freezing and heating (54). The immobilized water content of UIF samples increased and then decreased gradually as the ultrasonic power increased. The UIF-175-treated samples had the highest immobilized water contents because intracellular water was maintained or even increased. The size and dispersion of the ice crystals could be linked to this outcome. Large and extracellular ice crystal creation breaks the myofibrils to a large extent. Water generated from melting ice in extracellular areas was hardly reabsorbed by injured myofibrils after thawing. As a result, formerly immobilized water transformed to free water, which was then redistributed across the sarcoplasmic and extracellular regions. More intracellular water generated ice as the freezing rate increased, but it was unable to penetrate the extracellular areas (55). UIF-175 and UIF-190 treatments greatly decreased the freezing time and resulted in finer and more evenly distributed ice crystals, which would help keep the integrity of muscle tissue following thawing. As a result, water molecules were firmly bound in muscle tissue, which caused immobilized water to have a relatively low fluidity. Furthermore, the AF-treated samples had the largest free water contents, leading to a reduction in fish elasticity and an enlargement of thawing loss, which was in agreement with the TPA and thawing loss results described above. The UIF-175-treated samples had the lowest free water contents, which were more intimately connected with muscle organization than the other samples, resulting in less exudate (thawing loss).

Magnetic resonance imaging allows visualization of the spatial and interior morphology and molecular distribution of moisture in marine fish. The area with comparatively high proton density is shown in red, while the area with relatively low proton density is shown in blue (56). The color of the AF-treated samples was bluer than that of the other samples (Figure 6B), indicating that the AF-treated samples showed more extensive degradation and disintegration of the myofibrils. The UIF-treated samples contained more red regions than the AF- and IF-treated samples, implying that the samples were preserved in quality during the freezing process under ultrasound treatments. It is worth mentioning that the MRI findings were in line with the LF-NMR relaxation time changes.

### ATP-Related Compounds and *K*-Value

**Figure 7:**
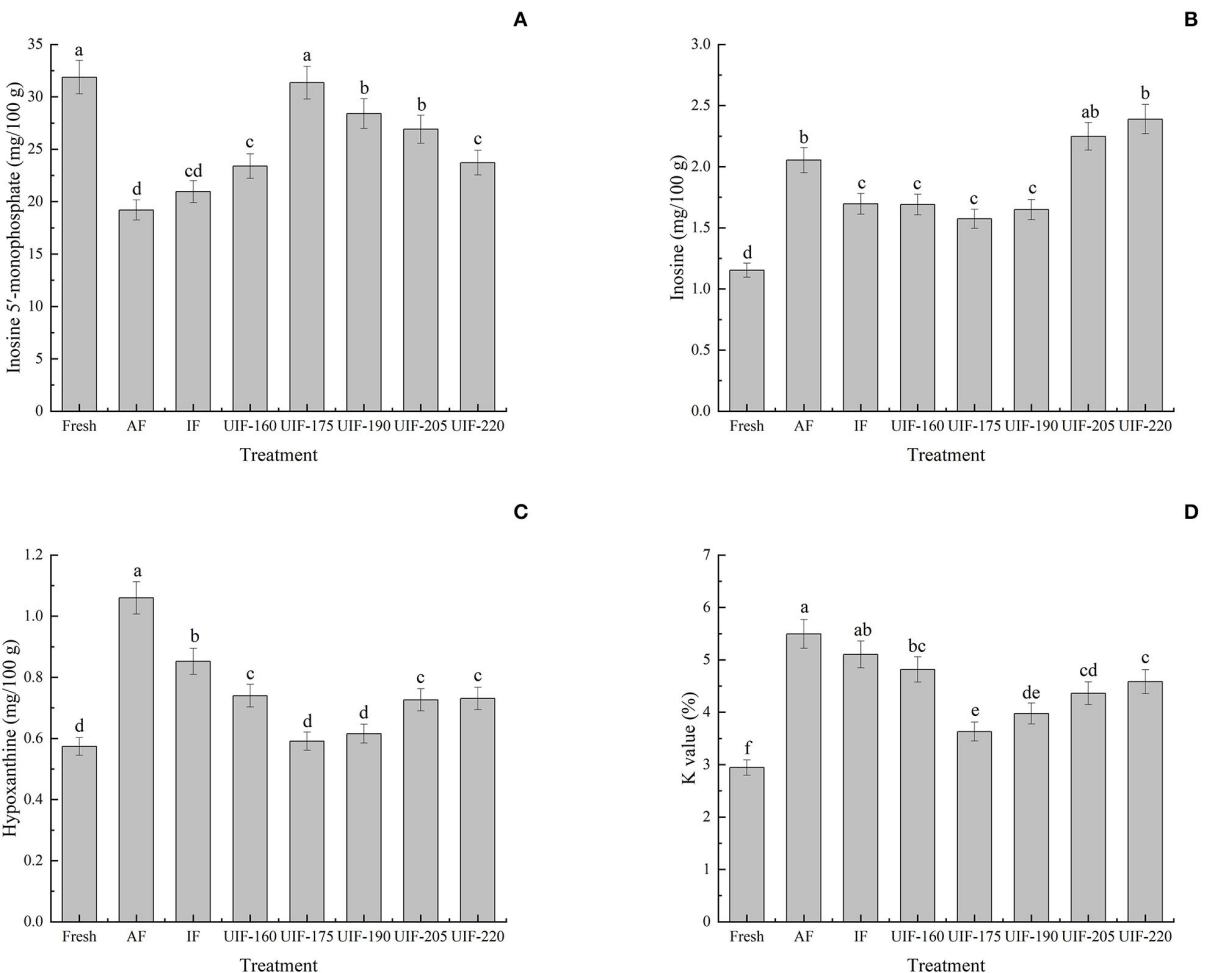
Influence of different freezing treatments on the inosine 5′-monophosphate (IMP) **(A)**, inosine (HxR) **(B)**, hypoxanthine (Hx) **(C)**, and K values **(D)** of the large yellow croaker. The results are average ± standard deviation (*n* = 3).

ATP degradation is one of the most important biochemical changes in the post-mortem muscle of fish and shellfish. This process has long been recognized as an accurate way to evaluate the freshness of fish and shellfish products. Not only are ATP-related compounds and their derived indicators (e.g., *K*-value) commonly used for freshness estimation, but they are also implicated in variations in the flavor quality of aquatic goods (57, 58). The umami flavor (a pleasant flavor) of fish and shellfish is connected with IMP, but the accumulation of Hx and HxR negatively affects the flavor because of their off-odor and bitterness (59). The initial IMP concentration in the fresh sample was 31.87 ± 1.59 mg/100 g (Figure 7A). And the concentration of IMP in all frozen samples was reduced in the presence of autolysis enzymes (60). The samples treated with UIF were frozen at an increased rate to preserve the structure and quality of the muscle and decrease the deterioration of IMP. The IMP concentration first increased and then declined with increasing ultrasonic power. The UIF-175-treated samples had the highest IMP concentrations compared to other samples, exhibiting an increase of 63.29 and 49.52% in comparison with AF- and IF-treated samples, respectively (*p* < 0.05).

Regarding the variations of HxR and Hx (Figures 7B,C), they were 1.15 ± 0.06 and 0.57 ± 0.03 mg/100 g in the fresh samples, respectively. The HxR and Hx contents in all samples increased progressively throughout the freeing process due to the degradation of ATP-related compounds, which led to the decline to a certain extent of fish quality during the freezing process. Furthermore, the HxR and Hx contents displayed comparable trends with a decrease at first and then had an increase in all samples as ultrasonic power increasing. The UIF-175-treated samples had the lowest HxR and Hx concentrations compared to other samples, exhibiting a decrease of 23.34 and 44.25% compared to the AF-treated samples (*p* < 0.05), respectively. While the UIF-205- and UIF-220-treated samples had higher HxR content than the other frozen samples as the collapse of cavitation bubbles during ultrasound treatment generates high-strength shock waves, turbulence, microstreaming, and shear forces, leading to extensive mechanical injury to the muscle (7). Furthermore, the freezing speed was delayed and big ice crystals in the fish muscle were generated because of the excessive heat created by high ultrasonic power (35).

The initial *K*-value in the fresh sample was 2.95 ± 0.15% (Figure 7D). It was evident that the UIF reduced the *K*-value and the UIF-175-treated samples had lower *K*-values than those of other samples (*p* < 0.05). Shi et al. (61) stated that the *K*-value of fish with ultrasound at 0.38 W/cm^2^ was smaller than that of fish without ultrasonic treatment (*P* < 0.01). The deterioration of ATP is primarily because of the action of phosphorylase and cathepsin. Chemat et al. proposed that power ultrasound can induce cavitation and that both high temperature and high pressure generated by the rupture of cavitation bubbles can result in a reduction in enzyme activity (62). This offers a rational explanation for the decrease of *K*-value by power ultrasound.

### Total Volatile Base Nitrogen

**Figure 8:**
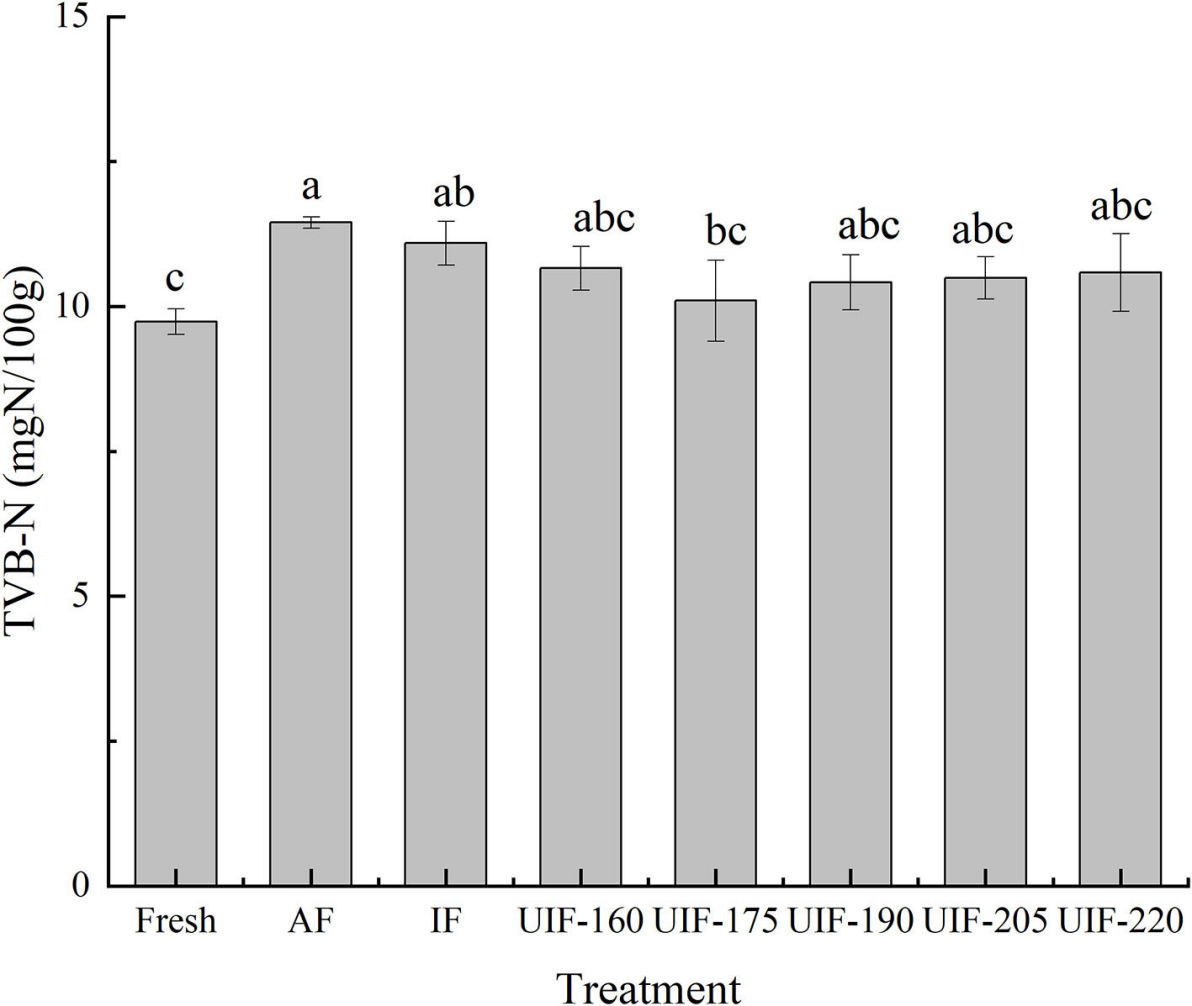
Influence of different freezing treatments on the TVB-N of the large yellow croaker. The results are average ± standard deviation (*n* = 3).

Fresh samples had an initial TVB-N of 9.74 ± 0.22 mg N/100 g (Figure 8). The UIF-treated samples had lower TVB-N levels than AF- (11.45 ± 0.10 mg N/100 g) and IF- (11.09 ± 0.38 mg N/100 g) treated samples. Vacha et al. reported that AF and IF treatments could form large ice crystals by slowly freezing to puncture the cells, adding the quantity of water outside cell space, and causing nutrient leakage, which provides a positive culture environment for microorganisms and results in an increase of TVB-N in fish (63). Besides, the TVB-N value of samples frozen by UIF-175 (10.10 ± 0.70 mg N/100 g) was lower than the samples frozen by other ultrasonic treatments and was closest to that of fresh samples, suggesting that rapid freezing can diminish the generation of TVB-N in fish samples. But no significant differences were observed among the samples frozen by different ultrasonic powers (*p* > 0.05). The results indicated that the samples frozen by UIF had a higher freshness than the other frozen samples. Sun et al. (37) discovered similar outcomes that the UIF treatment was more efficient in retarding the increase in TVB-N during storage in comparison with AF and IF treatments.

### TBARS Value

TBARS value is one of the indexes used to measure lipid oxidation in fish and fish products. It is used to quantify the secondary oxidation products from the degradation of polyunsaturated fatty acids. These secondary byproducts generate unpleasant tastes and rancid flavors in fat- and protein-rich foods (64). Although biochemical reactions are retarded to a great extent under freezing conditions, some non-negligible oxidative degradation still occurs.

**Figure 9:**
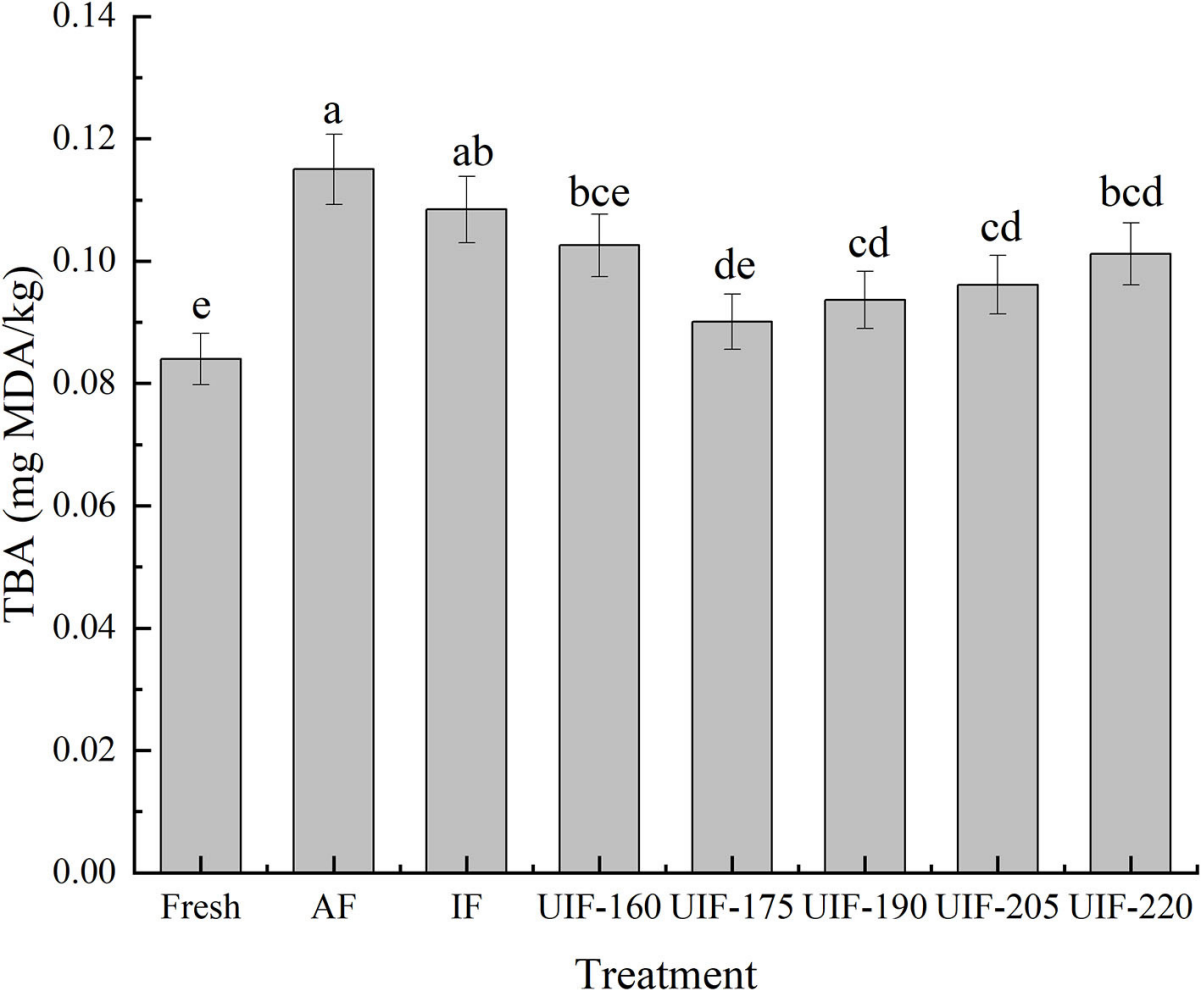
Influence of different freezing treatments on the thiobarbituric acid reactive substances (TBARS) of large yellow croaker. The results are average ± standard deviation (*n* = 3).

The TBARS values of all samples increased during the freezing process (Figure 9), which indicated that the lipid oxidation increased during the freezing process. This could be attributed to the development of ice crystals observed during the freezing process that destroyed the muscle cells and activated the release of oxidation precursors to promote the oxidation reaction (65). The TBARS value of fresh samples was ~0.08 mg MDA/kg. The TBARS of AF- and IF-treated samples were larger than those of UIF-treated samples. A significant difference was observed in TBARS values among the AF-treated samples and ultrasonic-treated samples (*p* < 0.05). This could be due to the extensive muscle damages that occurred in the AF- and IF-treated samples that accelerated the oxidation reaction. It can also be observed that the TBARS values obtained in UIF-175-treated samples (~0.09 mg MDA/kg) were lower compared with other samples. The UIF produced small and uniform ice crystals in muscle tissue may be the major reason for a lower extent of lipid oxidation. In addition, UIF could inhibit the lipids oxidation by decreasing lipase, phospholipase, and lipoxygenase activities (66).

### FAAs Analysis

The changes of FAAs in large yellow croaker on the fresh and different freezing treatments are presented in Table 2. FAAs have a crucial role in the flavor development of aquatic products because they can give umami, sweetness, and bitterness, among other taste characteristics (67). Large yellow croaker had a low histidine level of <5 mg/100 g, which was in agreement with the findings of Wang et al. (68) and Dou et al. (69). In addition, the histidine content in large yellow croaker was much lower than that in other fish, such as farmed Japanese sea bass (18.15 mg/100 g) (24), silver carp (36.5 mg/100 g) (70), and grass carp (178.4 mg/100 g) (59). The low content of histidine that serves as a bitter FAA with the lowest threshold, was probably another essential factor contributing to the delicious taste of the large yellow croaker. A significant difference was found between a fresh sample and frozen samples in total FAA contents (Table 3). Beklevik et al. (71) proposed that the decline in total FAAs might be associated with the loss of water-soluble FAAs through the thawing process. As previously stated, fast freezing greatly decreased the thawing loss of fish samples, which can explain the fact that UIF-175-treated samples showed the highest total FAA contents. A total of 16 FAAs were identified in the fresh fish samples. Among them, glutamic acid, glycine, alanine, and lysine were the dominant ingredients accounting for 10.43, 7.75, 25.86, and 15.42 % of the total FAAs, respectively. Ozden (72) stated that aspartic acid, glutamic acid, alanine, and glycine played a positive role in providing umami-sweetness taste of aquatic products. UIF-175-treated samples displayed higher contents of glutamic acid, glycine, and alanine with the values of 21.49 ± 0.33, 13.66 ± 0.08, and 29.61 ± 0.28 mg/100 g, respectively. At the same time, the decreases of 12.84–59.38, 0.88–39.53, and 4.56–26.38% for glutamic acid, glycine, and alanine in other freezing samples were exhibited in comparison with UIF-175-treated samples. Besides, the increase of aspartic acid and glycine was shown compared to fresh samples, but the contents of each sample presented fluctuating changes with no apparent effect. The results discussed above indicated the UIF at different power levels, especially for UIF-175, helped maintain the flavor characteristics of the large yellow croaker.

**Table 3:** Influence of different freezing treatments on the free amino acids (FAAs) content (mg/100 g) of the large yellow croaker.

**Samples**	**FAAs**								
	**Asp**	**Thr**	**Ser**	**Glu**	**Gly**	**Ala**	**Val**	**Met**	
Fresh	1.07 ± 0.01^f^	2.79 ± 0.03^g^	3.54 ± 0.03^f^	10.24 ± 0.06^f^	7.61 ± 0.02^f^	25.39 ± 0.03^h^	6.26 ± 0.14^d^	2.81 ± 0.37^d^	
AF	2.09 ± 0.07^c^	5.15 ± 0.08^d^	6.29 ±0.04^d^	8.73 ± 0.27^c^	8.26 ± 0.06^e^	22.65 ± 0.01^f^	11.25 ± 0.04^a^	5.84 ± 0.04^a^	
IF	2.57 ± 0.07^b^	6.37 ± 0.02^b^	7.27 ±0.04^b^	15.08 ± 0.15^a^	10.25 ± 0.03^c^	26.42 ± 0.10^e^	8.27 ± 0.05^b^	4.74 ± 0.04^b^	
UIF-160	2.52 ± 0.06^b^	5.61 ± 0.02^c^	6.51 ±0.02^c^	15.80 ± 0.03^d^	13.54 ±0.01^b^	27.86 ± 0.05^b^	7.61 ± 0.01^c^	4.76 ± 0.15^b^	
UIF-175	1.97 ± 0.05^d^	5.20 ± 0.08^d^	6.51 ± 0.07^c^	21.49 ± 0.33^b^	13.66 ± 0.08^d^	29.61 ± 0.28^d^	6.28 ± 0.53^a^	5.69 ± 0.55^a^	
UIF-190	1.28 ± 0.05^e^	3.88 ± 0.05^f^	5.65 ± 0.14^e^	18.73 ± 0.20^e^	13.46 ± 0.08^f^	28.04 ± 0.25^g^	8.42 ± 0.09^b^	4.79 ± 0.55^b^	
UIF-205	1.91 ± 0.02^d^	4.60 ± 0.03^e^	6.26 ± 0.01^d^	15.67 ± 2.26^d^	10.67 ± 0.05^b^	21.80 ± 0.18^c^	6.75 ± 0.48^d^	3.49 ± 0.45^c^	
UIF-220	3.10 ± 0.07^a^	7.10 ± 0.05^a^	13.81 ± 0.10^a^	16.34 ± 0.18^d^	10.71 ± 0.10^a^	28.26 ± 0.09^a^	4.59 ± 0.01^e^	2.93 ± 0.03^cd^	
	**Ile**	**Leu**	**Tyr**	**Phe**	**Lys**	**His**	**Arg**	**Pro**	**Total**
Fresh	4.31± 0.01^f^	7.31 ± 0.02^f^	3.10 ± 0. 01^c^	3.41 ± 0.01^de^	15.14 ± 0.03^b^	2.57 ± 0.02^b^	0.02 ± 0.01^d^	2.64 ± 0.61^e^	98.18 ± 0.70^f^
AF	8.76 ± 0.11^b^	13.05 ± 0.37^b^	6.70 ± 0.41^a^	6.53 ± 0.69^ab^	7.68 ± 0.51^e^	4.20 ± 0.25^b^	0.43 ± 0.01^d^	6.44 ± 0.33^ab^	123.63 ± 2.28^d^
IF	6.15± 0.04^c^	9.92 ± 0.21^c^	6.57 ± 0.50^a^	5.52 ± 0.67^bc^	26.05 ± 0.63^a^	4.32 ± 5.49^a^	0.30 ± 0.00^b^	6.69 ± 0.09^a^	146.53 ± 7.88^b^
UIF-160	5.57 ± 0.01^d^	9.10 ± 0.08^d^	5.94 ± 0.38^a^	4.52 ± 0.51^cd^	9.02 ± 0.51^d^	4.08 ± 0.19^b^	0.12 ± 0.01^d^	4.70 ± 0.19^d^	127.15 ± 1.25^d^
UIF-175	9.12 ± 0.10^a^	13.70 ± 0.39^a^	6.69 ± 0.52^a^	7.12 ± 0.80^a^	9.56 ± 0.58^d^	4.59 ± 0.28^b^	0.09 ± 0.01^d^	6.80 ± 0.11^a^	147.98 ± 3.55^c^
UIF-190	6.28 ± 0.10^c^	10.28 ± 0.17^c^	6.43 ± 0.09^a^	5.43 ± 0.05^bc^	14.25 ± 0.22^c^	4.27 ± 0.09^b^	0.22 ± 0.01^d^	5.72 ± 0.56^bc^	136.89 ± 1.05^e^
UIF-205	5.28 ± 0.02^e^	8.50 ± 0.02^e^	4.56 ± 0.07^b^	3.95 ± 0.34^d^	15.67 ± 0.07^b^	3.93 ± 0.02^b^	0.47 ± 0.01^a^	4.90 ± 0.03^d^	118.42 ± 2.56^d^
UIF-220	3.42 ± 0.01^g^	5.45 ± 0.02^g^	4.48 ± 0.15^b^	2.78 ± 0.32^e^	14.13 ± 0.02^c^	4.34 ± 0.02^ab^	0.17 ± 0.01^c^	5.25 ± 0.40^cd^	126.86 ± 0.57^a^

*The means in the same column with different superscript letters differ significantly (p < 0.05). The results are average ± standard deviation (n = 3)*.

**Figure 10:**
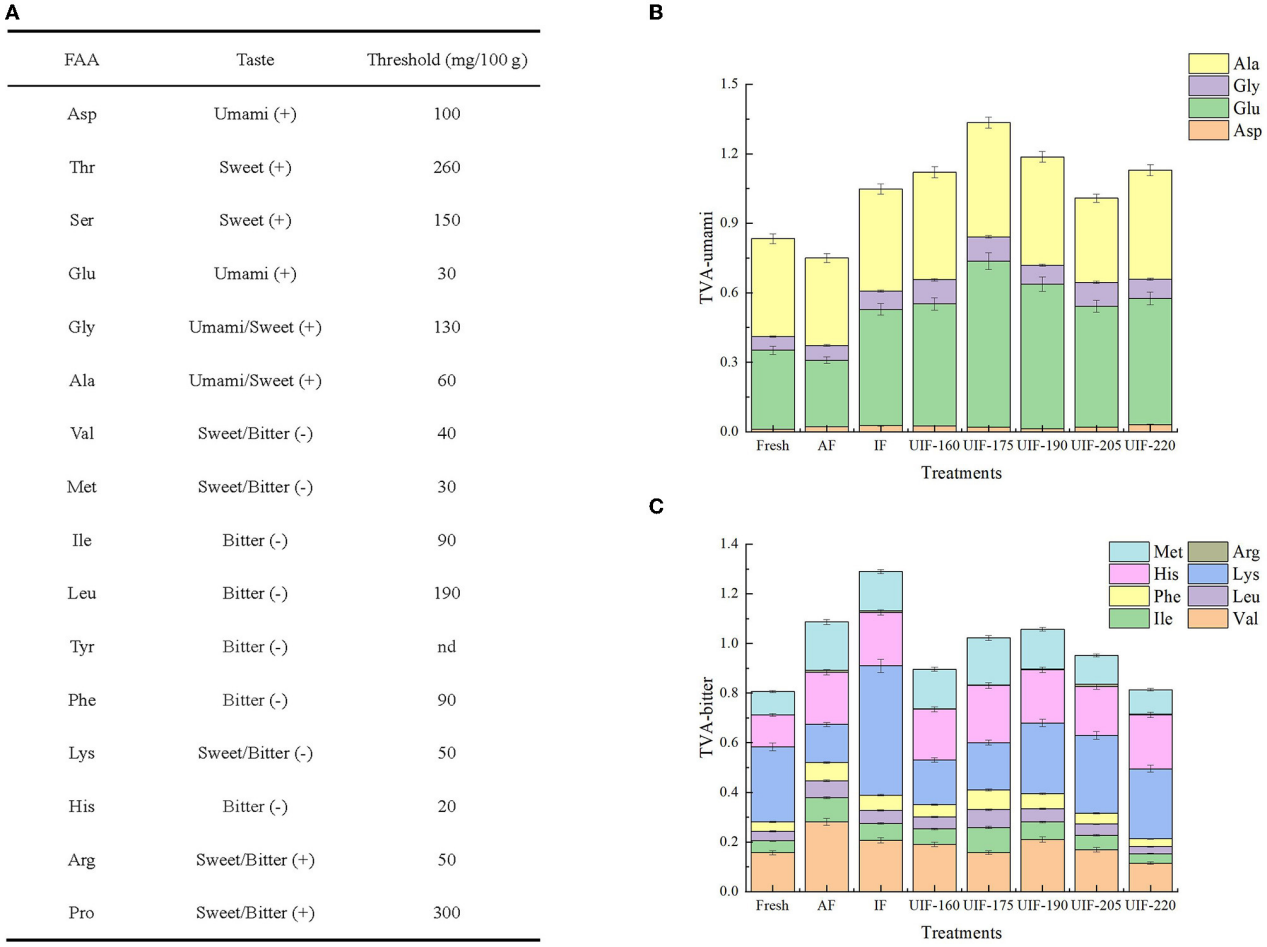
The thresholds of different amino acids taste **(A)**, changes in umami-TVA **(B)**, and bitterness-TVA **(C)** of fresh and frozen large yellow croaker under different freezing treatments.

Considering different thresholds of FAAs (Figure 10A), taste active value (TAV) was obtained according to Chen and Zhang (73) to further reflect the influence of different freezing treatments on flavor characteristics of frozen large yellow croaker. The umami-TAV obtained by aspartic, glutamic, glycine, and alanine were mostly high in all samples after the freezing process (Figure 10B). Among the UIF-treated samples, the umami-TAV values were higher than those of the AF and IF treatments at lower ultrasonic power, but showed a decrease at higher ultrasonic power. UIF-175-treated samples revealed the highest umami-TAV values. The total value of bitterness-TVA in UIF- (UIF-160, 175, 190, 205, and 220) treated samples were 17.56, 5.97, 2.72, 12.43, and 25.19% lower than that in AF-treated samples, and 30.53, 20.77, 18.03, 26.22, and 36.96% lower than that in IF-treated samples, respectively (Figure 10C). The UIF promoted the accumulation of umami-taste FAAs while reducing bitterness-taste accumulation.

## Conclusions

The influence of UIF at various powers on changes in the freezing speed, quality characteristics, and microstructure of the large yellow croaker were investigated in this work. The outcomes indicated that UIF at 175 W remarkably enhanced the freezing speed of the large yellow croaker. In addition, the UIF-175-treated samples had higher WHC and immobilized water contents, lower TVB-N and *K*-values, and better texture characteristics and uniformly distributed ice crystals microstructure, which were near to those of the fresh samples. FAA analysis revealed that UIF-175 treatment helped maintain the flavor of the large yellow croaker. But the freezing conditions did not show a significant effect on the color or pH values. Overall, increased freezing rate, improved quality attributes, and structural properties of large yellow croaker can be obtained with the application of suitable ultrasonic power.

## Data Availability Statement

The original contributions presented in the study are included in the article/supplementary material, further inquiries can be directed to the corresponding author/s.

## Author Contributions

XM: conceptualization, data curation, formal analysis, investigation, methodology, software, and writing—original draft. JM: conceptualization, data curation, investigation, methodology, project administration, validation, writing—original draft, and writing—review and editing. WQ: conceptualization, data curation, and writing—review and editing. JX: funding acquisition, methodology, project administration validation, and writing—review and editing. All authors contributed to the article and approved the submitted version.

## Funding

This research was funded by the China Agriculture Research System (CARS-47), the National Key R&D Program of China (2019YFD0901603), and the Shanghai Science and Technology Commission Platform Capacity Construction Project (19DZ2284000).

## Conflict of Interest

The authors declare that the research was conducted in the absence of any commercial or financial relationships that could be construed as a potential conflict of interest.

## Publisher's Note

All claims expressed in this article are solely those of the authors and do not necessarily represent those of their affiliated organizations, or those of the publisher, the editors and the reviewers. Any product that may be evaluated in this article, or claim that may be made by its manufacturer, is not guaranteed or endorsed by the publisher.
